# Early deviant behaviour as a dimension trait and endophenotype in schizophrenia

**DOI:** 10.4102/sajpsychiatry.v28i0.1747

**Published:** 2022-04-29

**Authors:** Johannes L. Roos, Carla Kotzé

**Affiliations:** 1 Department of Psychiatry, Faculty of Health Sciences, University of Pretoria, Pretoria, South Africa; 2 Weskoppies Psychiatric Hospital, Pretoria, South Africa

**Keywords:** early onset, deviant behaviour, schizophrenia, endophenotype, continuous symptom dimension

## Abstract

**Background:**

In psychiatry, there is still a lack of objective biological diagnostic measurements. It is important to investigate measurements or symptom dimensions that can inform diagnostic assessments and allow for a more personalised approach to patients.

**Aim:**

To discuss how early deviant behaviour (EDB) may be seen as a possible continuous symptom dimension trait and endophenotype in schizophrenia.

**Methods:**

Conducting a commentary review by highlighting some important findings from available literature.

**Results:**

Findings regarding EDB in schizophrenia in a South African genetic sample point towards EDB as a progressive subtype of schizophrenia, with very early onset of illness (even prior to the psychotic symptomatology) and a genetic form of illness.

**Conclusion:**

Valuable information can be gained by enquiring into EDB and viewing it as a continuous symptom dimension trait and endophenotype during the psychiatric diagnostic interview.

## Introduction

There is a lack of objective biological measurements to assess the ability of a genetic signature to predict or inform psychiatric practice.^1^ This generates a real opportunity for genetic findings to influence diagnostic and treatment processes to a more substantial degree than in other fields. Genetic support for schizophrenia as a discreet entity has been undermined by recent genetic findings. At least some of the genetic risks for schizophrenia also influence the risk for bipolar disorder (BD) and other mood disorders. Furthermore, there is a genetic overlap between schizophrenia and neurodevelopmental disorders.^2^

In an attempt to address difficulties with the investigation of mental illness, the Research Domain Criteria paradigm was introduced. Here, mental illness was conceptualised as dimensional extremes of normal human behaviour. Application of Research Domain Criteria to serious mental illness, such as schizophrenia, has been criticised as being conceptually flawed. Psychotic disorders are viewed as a spectrum of related disorders, with neurobiology and clinical phenotypes categorically distinct from normal human biology and function. These disorders are not easily understood using the Research Domain Criteria and research strategies that focus on discovery of aetiological factors have been supported.^3^

When we define phenotypes for mapping studies in genetics, two broad groups emerge, namely categorical traits and continuous traits. Categorical traits include Diagnostic and Statistical Manual (DSM) of Mental Disorders diagnostic categories. Continuous traits may include aspects like neurocognitive function, neuroanatomy and physiology, pharmacological response, neuroendocrine physiology, gene expression, personality and temperament.^4^ In one of these possible models, there is the assumption that every individual has a quantitative loading of a series of symptom dimensions (i.e. manic, psychotic, etc.). An assessment at the genetic level of these symptom dimensions can be used to specify a patient’s dysfunction. This can, then, be used to provide valuable information about the course of a disease and optimise treatment choices.^1^

Early deviant behaviour (EDB), where children exhibit aberrant behaviours that violate social norms, has been described as an endophenotype indicating early neurodevelopmental abnormalities and a high risk for schizophrenia.^5^ To deconstruct complex disorders like schizophrenia into smaller, objectively measurable parts, endophenotype is a valuable strategy to more suitably describe the way in which genes give rise to phenotypes.^6^

## Early deviant behaviour prior to onset of schizophrenia

Schizophrenia is seen as a neurodevelopmental disorder. The illness has its origin in the early childhood years.^7,8,9^ Retrospective and prospective studies of high-risk behaviour prior to developing schizophrenia show that EDB occurs in children who later develop schizophrenia.^10^ The deviant behaviour can be linked to early neurodevelopmental disorders.^11^ This may point in the direction of abnormalities in specific neural connections.

Prospective longitudinal studies of children who later developed schizophrenia showed that motor development was impaired and speech problems also occurred more commonly.^12^ The same authors emphasised on cognitive impairment and the children playing on their own. The EDB only occurs in a subgroup of schizophrenia patients and is connected to the later course of schizophrenia.^6,13^

Sobin et al. investigated EDB as an endophenotypic marker in genetic studies of schizophrenia.^5^ The group investigated was of European-caucasian origin and lived in the United States (US). The seven areas of EDB investigated in this group included social function, strange behaviour, unprovoked aggression, extreme anxiety, continuous heart sore, attention deficits and learning problems. The EDB had to be present before 10 years of age and on a constant basis. Sporadic or episodic occurrence combined with normal functioning, as well as problems starting after ten years of age, were not evaluated as the presence of EDB. The cut-off point of ten years was used to exclude behaviour connected to prepubertal hormonal changes. Furthermore, EDB connected to deprivation, abuse or seriously turmoiled families was also excluded, but the only comorbid conditions that were excluded were neurological disorders or brain damage.^5^

For a broader definition of the seven different areas of EDB, the reader is referred to the original text of Sobin et al.^5^ In our study, 60% of adult schizophrenia patients reported one or more area of EDB before the age of 10 years. This was a higher percentage than reported previously.^14^ Furthermore, they found a relationship between early social dysfunction and an earlier onset of schizophrenia. This confirmed previous findings in this regard.^15,16^ These findings are specifically important for genetic studies. As described by Scholtz et al., the seven types of deviant behaviour can be joined in three logical clinical clusters: ‘Social functioning impairment cluster (Social withdrawal/aggression/odd behaviour); Mood/Anxiety cluster (Extreme fears/chronic sadness); Cognitive impairment cluster (Attention impairment/learning disability)’.^17^

The study of phenotypic extremes with an early onset has been an important approach to the understanding of diseases across all medical disciplines.^18^ In early onset Alzheimer disease where multiple affected family trees were identified, presenilin was discovered.^19^ The accurate identification of onset of disease in complex illnesses is critical in genetic studies. It has been proposed that pre-schizophrenia EDB may point towards a developmental progressive subtype of schizophrenia.^20^

Sobin et al. proposed that EDB (and then specific social impairment) may point in the direction of illness onset and a genetic form of illness.^5^ In 2003, EDB was further investigated in a South African genetic sample for schizophrenia (SAGSS).^21^ Here, the researchers reported on a comparison study of EDB in the founder population and USA patients with schizophrenia and schizoaffective disorders (SAD).^22^ Important findings from the Sobin et al. studies regarding EDB included the following:

- Early deviant behaviour in the first 10 years of life occurred in about 68% of patients with lifetime diagnosis of schizophrenia and SAD. In daily clinical practice, about two thirds of this patient population reported EDB. These findings confirmed the results of previous studies.
- The group of patients with additional EDB differentiated a subgroup of patients where the illness started years before the onset of the full syndromal illness as described in the DSM classification.
- Early deviant behaviour is not included in the present psychiatric nosology. The types of EDB are connected to later syndrome characteristics. This may be an explanation of certain prodromal aspects of the later full syndrome of illness.^12,13,21^

Certain findings of more than 20 years of genetic research regarding EDB in the South Africa (SA) genetic sample for schizophrenia will now be discussed.

## Early deviant behaviour and specificity to schizophrenia

Scholtz et al. investigated non-psychotic EDB as an endophenotypic marker in BD, SAD and schizophrenia in a comparison study. They found that non-psychotic EDB was less prevalent in patients with BD than in patients with schizophrenia and SAD and it may therefore be a possible endophenotypic marker in schizophrenia and SAD. Patients with schizophrenia and SAD were also found to have significantly more impairment in social and cognitive functioning compared to patients with BD. A limitation of this study was that researchers did not collect data on psychosis in BD from the onset and it was found that 36% of patients with BD had a history of psychosis on retrospective analysis.^17^

There is an ongoing debate about the psychotic symptoms observed in schizophrenia and BD and whether this might indicate a diagnostic continuum with shared aetiology in these disorders.^23^ It has been argued that the psychosis continuum extends from BD to SAD, and at the other end, typical schizophrenia, reflecting increasing levels of severity.^24^

In a comprehensive analysis of determinants and characteristics of psychotic symptoms in BD, Van Bergen et al. showed that 83.8% of the patients presented a history of psychotic symptoms. The presence of psychotic symptoms in BD has been found to be associated with a predominantly manic course of the illness that is considered to be more severe. These findings support a differentiation in severity with BD based on psychosis vulnerability.^25^

The ‘Bipolar Disorder and Schizophrenia Working Group of the Psychiatric Genomics Consortium’ reported on a genomic dissection of BD and schizophrenia in 2018. Psychotic features in BD were determined to be a heritable trait. Genome-wide scans (GWS) loci for schizophrenia have a consistent direction of effect in psychotic features in BD. They demonstrated ‘the potential to study psychosis more directly to identify variants contributing to that symptom dimension’. A conclusion of their work was that after the genetics of multiple BD and schizophrenia cohorts were analysed it revealed ‘loci and polygenic risk scores that differentiate the clinical symptoms of these two highly correlated disorders’.^1^ The genetic complexity of these disorders contributed to the development of the polygenic risk score to measure the contribution of many small common variants to the risk of developing schizophrenia.^3^

## Genetic findings in Afrikaner schizophrenia patients where early deviant behaviour was included

Genome-wide linkage studies have suggested the location of a schizophrenia susceptibility locus at chromosome 22q11. This was also supported by further observation in patients with schizophrenia of a higher-than-expected frequency of 22q11 microdeletions. In individuals with 22q11 microdeletions approximately 20% – 30% develop schizophrenia or SAD during adolescence and adulthood. When further refinement of this locus was done to a region of ≈1.5 Mb, a high rate 22q11 microdeletion was also reported in childhood-onset schizophrenia. This is a severe form of schizophrenia with onset before age 13. It is likely that this 1.5-Mb region contains one or more genes that predispose to schizophrenia. The researchers provide evidence for a contribution of the Profline dehydrogenase 2 (PRODH2)/DiGeorge syndrome critical region gene 6 (DGCR6) locus in 22q11-associated schizophrenia, in three independent samples.^26^

A subgroup of adult schizophrenia patients from the SA genetic sample with early disease onset or EDB was examined. The reasoning behind this was that there was an apparent enrichment of 22q11 microdeletions in patients with childhood-onset schizophrenia. A further haplotype-based haplotype relative risk (HHRR) analysis was done, including only adult schizophrenia probands stratified according to the above-mentioned early-onset profiles. Three marker haplotypes of PRODH2 were identified. Using LD analysis in three independent non-deleted patient samples, the researchers provided strong evidence for an association between variation at the 22q11 locus and schizophrenia with early onset features.^17^

Proline is an amino acid and precursor of glutamate. Proline itself may serve as a direct modulator of glutamatergic transmission in the brain.^27^ An alternative model implicates PRODH2 in apoptosis.^28^ Apoptosis refers to cell death that occurs in a programmed manner. It is characterised by cellular condensation and is non-inflammatory or even anti-inflammatory through the induction of certain cytokines and generation of T regulatory cells. It thus differs from necrosis. These processes and their relationship to autoimmunity have been the subject of detailed investigations.^29^ Apoptosis plays a pivotal role in different processes, but also in morphogenesis and tissue remodelling during embryonic development.^30^

In 2012, researchers reported on dysregulation of DGCR6 and DGCR6L and the psychopathological outcomes in chromosome 22q11.2 deletion syndrome. Their investigation revealed that low expression levels of the vital genes DGCR6 and DGCR6L are associated with the observed variability in anxiety disorders in children with 22q11 deletion syndrome. It was hypothesised that this expression pattern could be used to predict anxiety and the development of associated schizophrenia. Further studies were recommended to confirm the use of this pattern as a biomarker.^31^ The variability in symptoms seen in individuals with 22q11 deletion syndrome is likely controlled by complex epigenetic mechanisms that, when disrupted, lead to dosage aberration rather than simple reduction in gene expression.^31^

## Phenotypic features where de novo gene mutations occurred in schizophrenia and the role of early deviant behaviour

Sporadic cases of schizophrenia may have de novo mutations, a genetic alteration that is present for the first time in one family member. This is the result of a mutation in a ovum or sperm of one of the parents or in the embryo itself and may reveal genes disrupted in schizophrenia.^32,33,34^ In a study that investigated the phenotypic characteristics of schizophrenia patients from an Afrikaner cohort it was found that two groups had de novo mutational status and a third group had no detectable mutational status. This study further also showed that those individuals with de novo copy number variants (CNVs) and single nucleotide variants (SNVs) had older parents at birth and worse functional outcomes than those patients without these mutations. Functional outcomes in patients with schizophrenia together with a history of EDB were considered as having a higher predictive value for underlying genetic vulnerability. Early deviant behaviour may be an early clinical manifestation that represents underlying premorbid neurodevelopmental pathology.^35^

## Advancing paternal age and poor social functioning prior to schizophrenia onset

Liebenberg et al. investigated the relationship between paternal age and EDB in the first 10 years of life, as well as longer-term functional outcome in sporadic Afrikaner founder population cases of schizophrenia. The paternal age at birth was found to have a significant negative relationship with EDB and social dysfunction in interpersonal relationships later in life.^36^ Paternal age-related schizophrenia is defined as patients with schizophrenia who have no family history of schizophrenia or psychosis and whose fathers’ age at birth was 35 years or older.^37^

The evidence reviewed support the general conclusion that individuals with schizophrenia have more social deficits prior to the onset of psychotic symptoms when compared to controls. Social withdrawal is not specific to schizophrenia at any age when compared with depression, anxiety or BD. The evaluation of social behaviour in combination with other risk factors may improve early identification of individuals at risk for development of psychosis.^38^ Children who score high on aggression and withdrawal are particularly at risk for later psychiatric illness, including psychosis.^39^ Psychosocial interventions can focus on these at risk individuals, especially when there is the additional risk of cannabis use which will be discussed in the following section.

## Cannabis use

In a cohort studied by Roos et al., more male schizophrenia patients than female were heavy users of cannabis. It was estimated that 25% of female patients with schizophrenia used cannabis, whilst this figure was approximately 50% in the male group. The youngest age of schizophrenia onset was found in males with severe EDB (two or more forms of EDB) and when they used cannabis the mean age of schizophrenia onset was 18.4 years. It was concluded that in males with severe EDB, cannabis can trigger the onset of psychosis and can contribute to a worse prognosis.^40^

In a more recent study, French et al. showed that cannabis use in early adolescence moderates the association between the genetic risk for schizophrenia and cortical maturation amongst male individuals. Experience-driven plasticity and related neuropil growth can increase cortical thickness, whilst testosterone-induced restructuring of neuropil can decrease cortical thickness in male adolescents. These cortical maturation processes have been implicated in mediating the link between cannabis use and liability to schizophrenia.^41^

Here, it may be necessary to mention the role of epigenetic risk factors in schizophrenia. The majority of genetic variants associated with schizophrenia are located outside gene-coding regions, suggesting a role for gene regulatory mechanisms. This regulation is a dynamic process across the lifespan. It is at the interface of genetics and the environmental epigenetic processes. It can be described as a biochemical record of environmental effects.^42^ By combining DNA methylation, histone modification and other epigenetic markers, provision can be made for a controllable and highly precise means to regulate the genome.^43^

There are two important reasons to study the deficits that are present in individuals before they develop psychotic symptoms. The specific nature of the pathological processes that lead to pre-psychotic schizophrenia is as still unclear, as is the point in development when these pathologies emerge. Findings may bring us closer to the specific nature of the pathological processes that lead to psychosis. Deficits that are sensitive and specific predictors of later psychosis, can offer valuable insight into the underlying pathologies involved in schizophrenia, including specific genetic and environmental liabilities.^44^ The level of social functioning achieved prior to psychosis onset is also an important predictor of post-onset symptom severity, cognitive deficits and overall level of functioning.^45^

## Childhood-onset schizophrenia and early deviant behaviour

The most common time for the onset of schizophrenia is from late adolescence to early adulthood, but it can also occur in children.^46^ Childhood-onset schizophrenia was a controversial diagnosis for many years. It was only clearly differentiated from autistic spectrum disorder by the landmark study by Kolvin et al. in 1971.^47^ Since 1990, a study of childhood-onset schizophrenia (onset ≤ 12 years) has been ongoing at the National Institute of Mental Health. This study found that there is biological and clinical continuity between schizophrenia with onset in childhood and in adulthood.^48^

In a descriptive study in 2009, Maydell et al. reported on the clinical characteristics and premorbid variables in childhood-onset schizophrenia of 12 cases from the South African genetic sample. A limitation of this study was the low number of patients included; however, childhood-onset schizophrenia is a rare entity. Of note was the finding of non-psychotic deviant behaviour in these 12 patients with childhood schizophrenia. All patients (100%) had EDB and, when analysed further, this included social functioning impairment (100%), mood and anxiety problems (58%) and cognitive impairment (58%). In this childhood-onset schizophrenia sample, the EDB was much higher compared to the adult South African genetic sample. In keeping with available literature, the authors concluded that EDB may be used as an endophenotypic marker for schizophrenia genetic studies.^49^

## Implications and recommendations

In the process of genetic schizophrenia research, researchers want to move closer to an objective biological measurement of schizophrenia. When researchers define phenotypes for mapping studies in genetics, two broad groups, categorical traits and continuous traits emerge.^4^ In the diagnostic categories, symptom dimensions (i.e. manic, psychotic) can be assessed at a genetic level.^1^ Researchers also have the endophenotype strategy, where a complex disorder like schizophrenia is deconstructed into smaller, objectively measurable parts to more aptly describe the way genes give rise to the phenotype. When researchers delve deeper into continuous traits, symptom dimensions and the endophenotype strategy, several core characteristics come to the fore. These include the following aspects: it can be assessed at a genetic level; disease onset; course and long-term outcome are described; deconstruction of complex disorders and objectively measurable parts are mentioned; as well as cognitive function.^1,4^

The findings regarding EDB and schizophrenia move close to the description of continuous traits, symptom dimensions and an endophenotype of schizophrenia. Early deviant behaviour may point towards a developmental progressive subtype of schizophrenia.^20^ In this subgroup of patients, the illness started years before the onset of the full-syndrome illness as described in the DSM.^21^ It was suggested that EDB points in the direction of illness onset and a genetic form of illness.^5^

Findings showed that EDB was less prevalent in BD compared to schizophrenia and SAD.^17^ Bipolar disorder patients had significantly less impairment in social and cognitive function as part of EDB.

The polygenic risk score has emphasised the genetic complexity of schizophrenia and the environmental interactions with genomic variants that increase risk.^3^ Strong evidence exists for an association between variation at the 22q11 locus and schizophrenia with early onset features and EDB.^26^ Evidence for a contribution of the PRODH2/DGCR6 locus in 22q11-associated schizophrenia was found and three marker haplotypes of PRODH2 were identified in the EDB schizophrenia group. More learning disabilities as part of EDB were found in subjects carrying de novo SNVs.^35^ Patients with de novo SNVs and CNVs had worse functional outcomes, and the combined consideration of the functional outcome scores and EDB was found to have a higher predictive value for underlying genetic vulnerability. A significant negative relationship between paternal age at birth and social dysfunction as part of EDB and interpersonal relationships later in life was found.^36^ The level of social functioning achieved prior to psychosis onset is also an important predictor of post-onset symptom severity, cognitive deficits and overall level of functioning.^45^ Male users of cannabis diagnosed with schizophrenia, with severe EDB, had the lowest mean age onset of illness, and EDB was linked to later outcome of the illness.^40^

It is recommended that patients presenting with EDB should be followed up closely and monitored for the onset of psychotic symptoms. This is even more important when they also present with other risk factors, such as cannabis use or cognitive impairments. Valuable information can be gained by enquiring into EDB and viewing it as a continuous symptom dimension trait and endophenotype during the psychiatric diagnostic interview.

## Limitations

The sample sizes in some of the studies discussed here were small. The EDB questionnaire is not a standardised instrument and information regarding EDB is mostly obtained retrospectively as reported by patients and parents. The validity of these methods can be questioned.

## Conclusion

It is important to study pre-psychotic deficits in schizophrenia, as the specific nature of the pathological processes that lead to psychosis is unclear, as well as the point in development when these pathologies emerge. Findings point towards EDB as a progressive subtype of schizophrenia. This form of schizophrenia has a very early onset (even prior to psychotic symptomatology) and is seen as a genetic form of illness. Further research into EDB as a continuous symptom dimension trait and endophenotype in schizophrenia may provide valuable information on timing and development of psychosis, and may offer greater insight into schizophrenia pathologies.
